# Reflections on Decisions Made on the Well-Established Use of Medicinal Products by EU Regulators and the ECJ

**DOI:** 10.3797/scipharm.1402-14

**Published:** 2014-03-24

**Authors:** John Joseph Borg, Andrea Laslop, Luca Pani, Romaldas Maciulaitis, Daniela Melchiorri

**Affiliations:** ^1^ Medicines Authority, 203 Level 3, Rue D’Argens, Gzira, GZR 1368, Malta; School of Pharmacy, University of Tor Vergata, Rome, Italy.; ^2^ BASG/AGES Medizinmarktaufsicht, Traisengasse 5, A-1200 Vienna, Austria.; ^3^ Italian Mediciens Agency, AIFA, Via del Tritone, 181, 00186 Rome, Italy.; ^4^ State Medicines Control Agency, Savanoriu, pr 220, LT-50148 Kaunas, Lithuania; Institute of Physiology and Pharmacology, Nephrology Clinic, Lithuanian Health Science University, A. Mickevičiaus g. 9, Kaunas LT-44307, Kaunas, Lithuania.; ^5^ Department of Physiology and Pharmacology, University of Rome “Sapienza”, Rome, Italy.

**Keywords:** Well-established use, Generics, Abridged applications, Regulatory affairs, Medicinal products

## Abstract

Background: In the European Union (EU), a medicinal product needs a marketing authorization (MA) to be placed on the market. The EU’s medicinal products’ legislative framework allows for a reduced application for medicines outside their data exclusivity. One such type of application is the well-established use (WEU) medicinal product application (i.e. bibliographic applications). Recently, these MA applications have been subject to arbitration procedures at the European Medicines Agency’s Committee for Medicinal Products for Human Use (CHMP) because of disagreements between member states during the authorisation process. This paper reflects on these cases and highlights their potential impact on future WEU applications.

Methods: Decisions adopted by the European Commission on WEU applications between 2009 and 2012 were identified from the EU Community Register on medicinal products for human use. Subsequently, decisions were reviewed to understand the potential serious risk to public health (PSRPH) that EU regulators raised during MA application procedures.

Results: Four decisions were adopted by the EU commission between 2009 and 2012. Three followed disagreements between member states on PSRPH grounds. One decision was the outcome of a centralised marketing authorisation application. Six key messages were identified from the four cases reviewed and presented.

Conclusion: A guideline on WEU to implement the technical specifications to fulfil Annex I of Directive 2001/83/EC for MA applications is not available. Thus, reflections on recent decisions on WEU applications provide scientific direction to the industry as well as the medicinal product regulators on the documentation required to successfully file and obtain a WEU MA.

## Introduction

### Novelty of the Work

In the EU, one type of medicinal product approval license is the one for old active substances (the Well-Established Use (WEU) application file). Unlike generic applications, to date there is no official EU guideline on the requirements for WEU products. The only guidance available arises from Commission decisions following arbitration at the European Medicines Agency’s Committee for Medicinal Products for Human Use (CHMP). This manuscript reviews these arbitration cases and their implications for the industry developing WEU medicinal products.

### Background

In the European Union (EU), a medicinal product needs a marketing authorization to be placed on the market. To obtain this authorisation, an application consisting of a dossier supporting the medicinal product’s quality, safety, and efficacy needs to be submitted to regulatory authorities. The dossier can either be a full stand-alone dossier consisting of the results of the studies (clinical and preclinical as well as quality) conducted by the prospective marketing authorisation holder usually for an “on patent” medicinal product (originator/innovator medicine) or a dossier consisting of a full package of quality documentation, but a reduced clinical and preclinical program. The latter is usually the case for medicines outside of their data exclusivity, such as generics, well-established use medicinal products, hybrid (mixed bibliographic applications), and biosimilar medicinal products. In the case of generics and biosimilars (“biogenerics”), applicants make a bridge to the reference medicinal product’s data supporting efficacy and safety contained within the reference medicinal product’s dossier.

Well-established use (WEU) medicinal product dossiers (i.e. bibliographic applications) need to fulfil the legislative requirements of Directive 2001/83/EC by showing that the medicinal product to be placed on the market is safe and efficacious as well as of good quality. This is demonstrated by the reference to appropriate bibliographic data, as well as a demonstration that the active substance(s) of the medicinal product have been in use within the EU for at least ten years, in terms of the conditions set out in Annex I of Directive 2001/83/EC [1], which requires evidence of recognised efficacy, together with an acceptable level of proven safety. The WEU route of obtaining a marketing authorisation in the EU is established by Article 10a of Directive 2001/83/EC. The WEU route has been used in certain cases as a legal basis for the registration of medicinal products when a reference medicinal product cannot be identified in the EU [2]. If a reference medicinal product can be identified, then prospective applicants should pursue a development program and submit an application file as a generic medicinal product. The WEU route for obtaining a marketing authorisation in the EU is not as common as the generic route. The EU Mutual Recognition Index product database lists 530 EU bibliographic procedures (i.e. WEU procedures) that have been processed (data as of 19 March 2013) [3]. Hence, although not as common as generic products (the MRI database lists 19,389 products (this value includes generics and mixed bibliographic as well as WEU applications (data as of 19 March 2013)), the WEU applications are not uncommon in the EU regulatory system. Some have recently been subject to referral procedures to the CHMP because of disagreements between member states during authorisation procedures. This paper reflects and should stimulate debate on recent decisions published by the European Commission on WEU applications. The examples are selected based on opinions adopted by the CHMP on a centralised procedure as well as the arbitration of decentralised and mutual recognition procedures, and the implications for Marketing Authorisation Holders are discussed.

## Methods

Decisions adopted by the European Commission on WEU applications between 2009 and 2012 were identified from the EU Community Register listing all medicinal products for human and veterinary use. See http://ec.europa.eu/health/documents/community-register/html/index_en.htm [4]. Adopted decisions contain the opinion of the CHMP upon which the decision was based. We reviewed the decisions and highlight their potential impact on future WEU applications.

## Results

Four decisions were adopted by the European Commission between 2009 and 2012. Three decisions were the result of scientific arbitration at the CHMP following disagreements between member states on Potential Serious Risk to Public Health grounds within a Mutual Recognition/Decentralised Procedure for a marketing autrhorisation. One decision was the outcome of a centralised marketing authorisation application.

### Case Reports

#### Ribavirin Capsules and Ribavirin Tablets, iQur Ltd (DK/H/1081/01 – 04/DC) [4, 5]

The above products were referred to the CHMP in December 2007 under Article 29(4) of Directive 2001/83/EC. On 24/10/2008, the European Commission published a decision refusing the marketing authorisation for the Ribavirin capsules and Ribavirin tablets. The grounds for refusal were based on considerations that the Applicant did not provide sufficient evidence for a systematic and documented use of the substance outside (a) clinical trials, (b) compassionate use (that is for products used following an official opinion given at CHMP for products under evaluation), and (c) named patient supply to demonstrate the well-established use of Ribavirin in the claimed indication in the European Economic Area (EEA). The Applicant also failed to sufficiently demonstrate the relevance to Ribavirin iQur of the bibliographic data submitted, which included products different from the medicinal product intended for marketing [5].

Drivers for this Commission decision included the fact that the bibliographical data submitted related to various Ribavirin-containing products (and therefore different formulations), which makes it very difficult to extrapolate the published data to Ribavirin iQur (this is of a special concern for clinical trials that may have used experimental formulations) intended for marketing, with regards to therapeutic indication, posology, duration of treatment, and patient population. Moreover, the literature provided to substantiate the WEU of Ribavirin in the claimed indication and was not adequate for at least ten years in the EEA. The evidence to meet the criteria of extensive clinical use outside clinical trials, compassionate use (that is for products used in practice following an official opinion given at CHMP for products under evaluation), and named patient supply in the claimed indication was not demonstrated. In fact, the bibliography relevant to the indication applied for and referred to by the Applicant consisted of pilot studies conducted in small cohorts of patients (mostly non-responders or relapsed patients) receiving the combination therapy with Interferon. The CHMP concluded that the application for Ribavirin iQur did not satisfy the requirement of the WEU legal basis and recommended the refusal of the granting of a marketing authorisation [5].

#### Dexamethasone Oral Solution, Alapis SA – EMEA/H/A-29/1308 [4, 6]

The above product was referred to the CHMP in May 2011 under Article 29(4) of Directive 2001/83/EC. On 24/10/2011, the European Commission published a decision allowing the granting of a marketing authorisation for Dexamethasone oral solution, Alapis SA. The reason the product was referred for arbitration was because some member states involved in the procedure considered that the literature data on Dexamethasone tablets that had been submitted could not be extrapolated to Dexamethasone oral solution without adequate bridging data. According to the member states who disagreed with the reference member state and other concerned member states, the safety and efficacy of Dexamethasone Alapis could not be established without this bridging data. The documentation submitted in the application file showed that Dexamethasone has been widely used in EU clinical practice for numerous decades for a number of indications [6]. Over 180 literature reports (Randomized Clinical Trials (RCTs); Reviews, Monographs (Martindale) etc.) were included in the application dossier. However, the vast majority of publications were with tablet formulations and a few with oral solution/syrups [6]. Literature data available on the various routes of administration for the same treatment show that Dexamethasone is equally effective via any route of administration and that Dexamethasone has a broad therapeutic index. However, as information on the composition of the Dexamethasone syrup, elixir, or oral solution investigated in the provided studies was missing in all the submitted articles, the issue on whether those data could be considered as sufficient to allow bridging of the data between the tablets and the oral formulation applied for was raised. Additional bridging data between the bibliographic data and the proposed pharmaceutical product were therefore requested [6]. The Applicant applied for a BCS (Biopharmaceutics Classification System)-based biowaiver according to the Appendix II of the “Guideline on the investigation of bioequivalence (CPMP/EWP/QWP/1401/98 Rev 1/Corr)” [7], In order to extrapolate efficacy and safety data from the bibliographic data in the application file to the oral solution formulation applied for, the Applicant provided data that showed that irrespective of the immediaterelease oral dosage formulation (tablet or solution), the bioavailability of Dexamethasone, in terms of extent and rate of absorption, was similar. This was done through 1) a parallel artificial membrane permeability assay (PAMPA) data study, 2) literature, and 3) dissolution data, demonstrating that the Dexamethasone drug substance has bio-pharmaceutical characteristics of BCS Class I/III, and that the excipients have no adverse effects on bioavailability. The Applicant showed that Dexamethasone Alapis met all criteria for a BCS-based biowaiver [6]. This comprehensive package of literature data, supported by the requested data collected by the Applicant, enabled the CHMP to conclude on a positive benefit/risk balance for the product and recommend the marketing authorisation of Dexamethasone Alapis to the European Commission (EC).

#### Loraxin Vitabalans Oy (Loratadine, 10 mg Tablets) – EMEA/H/A-29/1325 [4, 8]

The above product was referred to the CHMP in December 2011 under Article 29(4) of Directive 2001/83/EC. On 20/12/2012, the European Commission published a decision refusing the marketing authorisation for Loraxin Vitabalans Oy, 10 mg tablets. The reason the product was referred for arbitration was because some member states considered that the literature data submitted could not ascertain the safety and efficacy of the medicinal product intended to be placed on the market. Although there was no issue with establishing that loratadine has been in WEU in the EU over the last decade, for most of the clinical trials cited by the Applicant supporting efficacy and safety, the medicinal product used (and therefore its formulation) within these studies was not clearly defined [8]. These studies included loratadine products in several strengths from 5 mg up to 40 mg. In these studies, pharmacokinetic parameters have been studied after a single dose as well as after repeated administration. The studies were conducted in children, healthy adult volunteers, and renally impaired patients [8]. In order to bridge the bibliographic data cited to Loraxin, the Applicant referred to pharmaceutical, pharmacokinetic, and clinical data. From a pharmaceutical perspective, the Applicant argued that the literature on the originator product is relevant for their product on the basis of the submitted pharmaceutical data. This was not upheld by the CHMP, who considered that since loratadine is not a BCS class I (high solubility-high permeability) or III (high solubility-low permeability), drug extrapolation based on the pharmaceutical data is not considered scientifically valid. The Applicant also submitted pharmacokinetic study data (Report V-808), which was evaluated by CHMP [8]. The inclusion of pharmacokinetic data in support of a WEU application is allowed, if it is intended to show the relevance of the literature used to demonstrate safety and efficacy with regards to the product concerned. The pharmacokinetic study provided to bridge Loraxin to the published literature and the AUCt and Cmax of Loraxin to the originator product Clarityn did not conform to the acceptance interval of 0.8–1.25 for the parent compound, loratadine. Based on the study results used to bridge Loraxin to the published literature, there is a potential for difference in exposure after Loraxin administration as compared to the product Clarityn, and the Marketing Authorisation Holder had not adequately justified why this potential difference in exposure would be unlikely to lead to a clinically significant difference in efficacy and/or safety. Searching the Pubmed database for loratadine retrieves many publications on Clarityn. Therefore, as the Applicant in this application had cited these studies, the data evaluated by the CHMP could have been classified as “a single source bibliographic data (i.e. carried out with reference product)” and in consequence this product failing the bioequivalence study could be considered as a failed generic medicinal product. In conclusion, the CHMP considered that the pharmaceutical, pharmacokinetic, and clinical data/documentation referred to by the Applicant was not sufficient to establish the relevance of the bibliographic data to the product applied for and no recommendation for a marketing authorisation should be expressed [8].

#### Orphacol; Laboratoires CTRS (Cell Therapies Research & Services) EMEA/H/C/1250 [4, 9]

Orphacol “cholic acid” was a designated orphan medicinal product [10] in the following indication: treatment of inborn errors in primary bile acid synthesis. In October 2009, Laboratoires CTRS submitted a centralised application procedure for a marketing authorisation for the following indications: treatment of inborn errors in primary bile acid synthesis due to 3β-Hydroxy-Δ5-C27-steroid oxidoreductase deficiency or Δ4-3-Oxo-steroid-5β-reductase deficiency in infants, children, and adolescents aged 1 month to 18 years and adults [9]. The application was a WEU application with bibliographic data supporting efficacy and safety. The application also referred to the existence of exceptional circumstances in accordance with Article 14(8) of the Regulation (EC) No 726/2004, due to the rarity of the conditions that the product intends to treat, as well as the inability to collect comprehensive information, because it would be contrary to medical ethics [11, 12]. On 16 December 2010, the CHMP recommended the granting of a marketing authorisation under exceptional circumstances in accordance with Article 14(8) of Regulation (EC) No 726/2004 [11]. The rationale behind the CHMP’s opinion was due to the position that the few patients reported in the literature were considered sufficiently representative of the EU patient population [9]. Since the collection of further data in this population is not feasible, the CHMP considered that it would be contrary to generally accepted principles of medical ethics to collect such information. However, the European Commission (EC) refused to grant the marketing authorisation based on the following reason: applications for a marketing authorisation must contain specifically and completely the particulars and documents as referred to in Articles 8(3), 10, 10a, 10b, or 11 and Annex I to Directive 2001/83/EC [1]. The Commission further explained that the requirement that marketing authorisation applications contain adequate data is fundamental to ensure the quality, efficacy, and safety of medicinal products [9]. Thus. the standard data requirements set out in Annex I of Directive 2001/83 need to be complied with. Therefore, the EC concluded that a WEU application supported by clinical studies based on exceptional circumstances was not legally compatible with the Directive. However, on 4 July 2013, the Commission was ordered by the European Court of Justice (ECJ) to reverse its decision to refuse to authorise Orphacol [9].

## Discussion

The WEU application is a type of application for the authorisation of medicinal products in the EU. Across the EU, more than 500 medicinal products (please note that we have grouped the products: i.e. per strength and pack size is reported as 1 product) have been approved through the legal basis of WEU. This makes the WEU route not an unusal procedure in relative terms and disagreements between member states during mutual recognition/decentralised procedures have resulted in a number of arbitrations at the CHMP over the last couple of years. The applicable guideline on the WEU application file is the European Commission’s Notice to Applicants. However, a specific guideline on WEU and the technical specifications to fulfil Annex I of Directive 2001/83/EC is not available. This does not mean that such a guideline is required or needs to be developed, as EU regulators have managed to handle many WEU application files over the years. Yet in this scenario, the review of cases at CHMP takes more prominence as it sets the scene of the current understanding of the WEU application and how applications will be assessed. The four cases reviewed in this paper are extremely interesting and each case enhances the understanding of what a WEU application should address. Key points identified (see conclusion) from the outcomes on these WEU applications are important for manufacturers of medicinal products intending to submit a WEU application to EU regulators so that they can submit a successful application. To the best of our knowledge, this is the first review of WEU applications referred for arbitration to the CHMP during the scientific evaluation of marketing authorisation applications since the CHMP was set up [11]. The aim of this review is to identify the challenges faced with WEU applications in order to help reduce unnecessary and avoidable delays in otherwise safe and effective medicines reaching the EU market. This will ultimately be of benefit to patients.

## Conclusion

The cases reviewed in this paper enhance the understanding of what a successful WEU marketing authorisation application should address.

The following key points have been identified from the four cases and should help the industry improve the quality of their submissions for marketing authorisations based on WEU in the EU:

• in order to fulfil the WEU legal basis, extensive clinical use outside clinical trials (this is only applicable for WEU orphan medicinal products [as a process introduced by the EU’s orphan legislation]), compassionate use (that is for products used following an official opinion given at CHMP for products under evaluation), and named patient supply, in the claimed indication, needs to be demonstrated.
• the inclusion of multi-source bibliographical data with various different formulations in the application file can make it very difficult to extrapolate the published data to the product applied for.
• the inclusion of single source bibliographical data (i.e. with only one formulation) makes it easier to extrapolate the published data to the product applied for, although in some cases a clinical study [only bioequivalence study allowed] showing the pharmacokinetic similarity might be required to bridge the bibliographical data.
• in a WEU application file, a bridge needs to be established between the literature and the product applied for. To be able to create this bridge, the applicant can use data from pharmacokinetic studies it has carried out. These studies could establish and support the bridge to the literature cited; furthermore, pharmacokinetic parameters should meet the acceptance criteria defined in the EU bioequivalence Note for Guidance.
• If scientifically justified, the use of the BCS-based biowaiver can also be applied in WEU applications and is not only scientifically valid for a generic application. The “Guideline on the investigation of bioequivalence (CPMP/PWP/EWP/1401/98 Rev 1/Corr)” Annex is useful to set what criteria are applicable for a BCS-based biowaiver [7].
• The European Court of Justice’s ruling on Orphacol has demonstrated that it is possible for a marketing authorisation to be granted under exceptional circumstances for an orphan medicinal product using the WEU legal basis where issues of public health need are identified [9, 13].

## Authors’ Statements

### Disclaimer

The views expressed in this article are the personal views of the authors and may not be used or quoted as being made on behalf of, or reflecting the position of, any national competent authority, the EMA, or one of its committees or working parties or any University.
